# Physicians’ prescribing behaviour and clinical practice patterns for allergic rhinitis management in Italy

**DOI:** 10.1186/s12948-020-00135-4

**Published:** 2020-11-03

**Authors:** Giovanni Passalacqua, Antonino Musarra, Gianenrico Senna, Jean Bousquet, Carmen Ferrara, Caterina Lonati, Giorgio Walter Canonica

**Affiliations:** 1 Allergy and Respiratory Diseases, IRCCS Policlinico San Martino, University of Genoa, Genoa, Italy; 2 Allergy Unit, National Healthcare System, Scilla, Reggio Calabria Italy; 3 Unità Operativa di Allergologia-Asma Center-Azienda Ospedaliera, Universitaria Integrata di Verona, Verona, Italy; 4 Comprehensive Allergy Center, Department of Dermatology and Allergy, Charité–Universitätsmedizin Berlin, Corporate Member of Freie Universität Berlin, Humboldt-Universität zu Berlin, and Berlin Institute of Health, Berlin, Germany; 5 Centre Hospitalier Universitaire de Montpellier, Montpellier, France; 6 MACVIA-France, Montpellier, France; 7 Mylan Italy s.r.l, Milan, Italy; 8 Center for Preclinical Research, Fondazione IRCCS Ca’ Granda Ospedale Maggiore Policlinico, via Pace 9, 20122 Milan, Italy; 9 Asthma & Allergy Clinic-Humanitas University & Research Hospital Milan, Milan, Italy

**Keywords:** Allergic rhinitis, Italy, Allergologists, General practitioners, ENT specialists, Pharmacological management, Undertreatment, Patient adherence

## Abstract

**Background:**

Despite availability of clinical guidelines, underdiagnosis, undertreatment, and poor adherence are still significant concerns in allergic rhinitis (AR) therapeutic management. We investigated clinical practice patterns and prescribing behavior of Italian healthcare professionals (HCPs) specialized in AR.

**Methods:**

One-hundred allergologists, 100 ear, nose and throat (ENT) specialists, and 150 general practitioners (GPs) were recruited. The survey assessed: socio-demographic, work experience, monthly caseload, prescription drivers. Next, HCPs were invited to retrospectively recover patients’ clinical data to investigate: AR clinical characteristics, therapy management, prescription patterns, patient adherence. Descriptive statistics, Chi square, One-Way analysis of variance, and Two-Way Analysis of Variance were performed.

**Results:**

Allergologists visited more AR patients (31% of monthly caseload) than ENTs (21%, p < 0.001), while GPs’ caseload was the lowest (6%). Clinical information of 2823 patients were retrieved of whom 1906 (67.5%) suffered from moderate/severe AR (discomfort score: 7.7 ± 1.3) and 917 (32.4%) from mild AR (5.7 ± 1.9). About one-third of mild patients had a discomfort score ≥ 7. Main prescription drivers were “effective on all symptoms” (54.3% patients) and “quick symptom relief” (47.8%), whereas minor drivers were “affordable price” (13.4%) and “refundable” (8.7%). The most prescribed drugs were antihistamines and intranasal corticosteroids (79% and 55% prescriptions), followed by fixed-dose-combination of intranasal azelastine/fluticasone (19%). Polytherapy was the most common treatment strategy (59.6%). HCPs’ believe that the majority of the patients was adherent to treatment (88% with score > 7).

**Conclusions:**

This survey describes the therapeutic approach adopted by Italian physicians to cope with AR and shows that HCPs underestimated AR severity and had a non-realistic perception of patients’ adherence. These findings suggest that further efforts are required to improve AR clinical management in Italy.

## Introduction

Allergic rhinitis (AR) is one of the most common disease affecting adults worldwide with increasing incidence and prevalence in almost all western countries [1–5]. Though it is not a serious condition, AR is widely accepted as a clinically relevant and disabling disorder accounting for a substantial burden of global morbidity [3, 6] and it is associated with considerable economic impact [7–9]. Indeed, patients with AR experience particularly bothersome symptoms which negatively affects their everyday activities and quality of sleep, ultimately leading to reduced quality of life (QoL) [3, 10–12] and impaired work and school performance [9, 13]. In asthmatics subjects, coexistent AR exacerbates severity of asthma [14, 15].

Despite international and national are continuously reviewed and updated to optimize patient care guidelines (e.g. Allergic Rhinitis and its Impact on Asthma, ARIA) [16–19], AR clinical management is still unsatisfactory, with high rates of underdiagnosis [12, 20, 21] and undertreatment [20–23]. Inadequate control of symptoms not only is associated with delayed medical examinations and patient preference for over-the-counter drugs, but it can also cause serious diseases such as nasal polyp development, acute and chronic sinusitis, and otitis media [23, 24]. Patients’ low adherence to therapy is an additional factor affecting achievement of proper symptom control [25–27].

In Italy, prevalence of AR has increased over the last 20 years from 16.8 to 25.8% [1, 28, 29]. A survey by Spinozzi and coworkers showed that over half of the patients recruited by general practitioners (GPs) experience symptoms which significantly impairs their daily/social life [30]. Strikingly, more than 25% of the interviewed subjects received no treatment despite the symptoms and 13.5% were inadequately treated. In addition, recent studies reported poor adherence to ARIA guidelines by Italian clinicians [31, 32].

The present research investigated the current clinical practice patterns and prescribing behaviour of Italian healthcare professionals (HCPs) specialized in AR management. Allergologists, ear, nose and throat (ENT) specialists, and GPs were asked to retrospectively recover clinical data of real-life cases to assess: (1) prescribing behaviour based on patients’ characteristics; (2) AR therapeutic management; (3) opinions toward patients’ adherence.

## Methods

### Design, HCPs’ recruitment, and data collection

We carried out a survey among a total of 350 Italian HCPs treating patients suffering from AR. The survey sample included 100 allergologists, 100 ENT specialists, and 150 GPs. HCPs were randomly selected from a national database. Exclusion criteria were: < 5 years of clinical practice, < 5 AR patients visited over the last month, participation to another market research in the previous 6 months. Recruitment was carried out via mail and it was planned to equally represent physicians from all Italian geographical macro-regions in each specialty area. The interviews were performed in April 2019 and data were collected through Computer Assisted Web Interviewing (CAWI) lasting 20 min.

The questionnaire used in the present research was designed based on findings from a systematic literature review and included two sections. The first section collected the HCPs’ socio-demographic data, such as age, gender, years of work experience, and number of monthly visited patients with AR. HCPs’ attitude toward relevant prescription drivers was likewise investigated (Additional file 1: Appendix S1).

In the second section of the survey, HCPs were invited to retrospectively recover clinical information of the patients they visited over the last month. The information retrieved included patients’ demographics, disease characteristics (disease symptoms, presence of concomitant asthma) and symptoms-related discomfort experienced by patients and disease severity (Additional file 1: Appendix S2). More specifically, HCPs were asked to allocate their patients into 2 classes of severity: (1) mild, when symptoms experienced by patients do not interrupt sleep or interfere with daytime activities; (2) moderate/severe, when symptoms cause significant difficulties with sleep and adversely affect daytime function [33]. Thereafter, therapy management, i.e. class of prescribed drugs, medication regimen, and follow-up intervals were investigated (Additional file 1: Appendix S3). Medication regimes included: (1) monotherapy, when a single drug was used; (2) concomitant polytherapy, in which different drugs were simultaneously used; (3) sequential polytherapy, in which the use of a specific drug was sequential to the use of another drug (i.e. drugs given one after the other); (4) polytherapy, in which different drugs were used, some to be taken continuously and other to be taken as-needed. Next, prescription drivers based on patients’ characteristics (Additional file 1: Appendix S4) were assessed. Physicians’ opinions about patient adherence to treatment was likewise explored (Additional file 1: Appendix S5). Finally, HCPs’ perception of AR economic burden (i.e. patients’ absenteeism from work and reduced productivity) was evaluated (Additional file 1: Appendix S6).

### Statistical analysis

Data were homogenously collected by means of a questionnaire including both multiple choice questions and Likert scale-based questions. A descriptive analysis was performed for all the evaluated variables, presenting the absolute frequencies in case of categorical variables and the mean with standard deviation in the case of the continuous variables. Mean ratings obtained from Likert-type scale-framed questions were used to investigate differences across study groups.

Differences in variable distributions across specialists were tested with Χ_2_ Chi square/or One Way analysis of variance (ANOVA) when appropriate. Kruskal–Wallis One Way Analysis of Variance on Ranks followed by Dunn’s post hoc test were likewise used. Two Way ANOVA was used to investigate significance between specialty area and patient assignment to the different classes of AR severity. Tukey’s post hoc test was used for pairwise multicomparison procedure.

A p < 0.05 was considered statistically significant. The data were analyzed using the statistics software SigmaPlot 11.0 (Systat Software, San Jose, CA, USA).

## Results

### Physicians’ sample characteristics and prescription drivers

Relevant characteristics of the 350 respondents are described in Table 1. With regard to AR caseload, allergologists visited more patients in the last month (a median of 40 patients, 31% of total caseload) than ENTs (21 patients, 21%) (p < 0.001), while GPs’ caseload was the lowest (18 patients, 6% of caseload). About half of the patients seen by allergologists (45%) and ENTs (42%) received a new diagnosis of AR, whereas 80% of the AR patients visited by GPs were already diagnosed (p < 0.001).

**Table 1:** HCPs’ characteristics

Characteristic	Whole sample	Allergologists	ENTs	GPs	p value
	N = 350	N = 100	N = 100	N = 150	
Age, years	56.9 ± 8.2	53.47 ± 10.91	55.60 ± 7.80	60.28 ± 5.40	*< 0.001*
Clinical experience, years	27.7 ± 9.4	24.48 ± 11.10	27.28 ± 8.65	30.14 ± 7.92	*< 0.001*
Geographic area					–
Northwest Italy	86 (24.5%)	25 (7.1%)	24 (6.8%)	37 (10.5%)	
Northeast Italy	60 (17.1%)	14 (4%)	19 (5.4%)	27 (7.7%)	
Central	78 (22.2%)	25 (7.1%)	21 (6%)	32 (9.1%)	
South and Insular Italy	126 (36%)	36 (10.2%)	36 (10.2%)	54 (15.4%)	
Patients volume/month	210 [100–400]	150 [100–247]	150 [100–300]	400 [300–500]	*< 0.001*
AR patients volume/month	20 [12–50]	40 [20–80]	21 [14–50]	18 [10–30]	*< 0.001*
% of new diagnosis/month	33.4 ± 25.3	45.3 ± 24.7	42.1 ± 25.4	19.6 ± 18.0	*< 0.001*
Prescription drivers					
Quick symptom relief	8.9 ± 1.2	9.0 ± 1.2	8.9 ± 1.3	8.8 ± 1.3	0.490
Effective with few drugs	8.8 ± 1.3	9.9 ± 1.0	8.7 ± 1.5	8.7 ± 1.4	0.219
Effective on all AR symptoms	9.1 ± 1.1	9.4 ± 0.9	9.1 ± 1.1	9.1 ± 1.3	0.101
Sustained efficacy	8.9 ± 1.1	8.9 ± 1.1	9.0 ± 1.2	8.9 ± 1.3	0.819
Few/no side effects	9.1 ± 1.0	9.3 ± 1.1	9.2 ± 1.1	9.1 ± 1.0	0.373
Supported by scientific literature	8.6 ± 1.4	8.9 ± 1.5	8.7 ± 1.3	8.5 ± 1.4*	*0.043*
Easy to take	8.5 ± 1.4	8.7 ± 1.3	8.4 ± 1.9	8.6 ± 1.3	0.434
Increased patient adherence	8.8 ± 1.2	8.9 ± 1.2	8.9 ± 1.4	8.8 ± 1.1	0.794
Refundable	6.8 ± 2.5	7.5 ± 2.6	5.7 ± 2.9*#	7.2 ± 2.1	*< 0.001*
Affordable price	8.1 ± 1.7	8.4 ± 1.6	7.9 ± 0.2	8.2 ± 0.1	0.128

Data are expressed as mean ± SD, median [25–75] or N (%). Chi squared test was used to investigate differences in the observed frequencies across specialty area. In case of discrete variables, differences across specialty area were evaluated using one way analysis of variance followed by Tukey test for all pairwise multiple comparison procedure or Kruskal–Wallis one way analysis of variance on ranks followed by Dunn’s post hoc test: p < 0.05: * vs allergologists, # vs GPs

All the prescription drivers presented through the questionnaire (Additional file 1: Appendix S1) were rated high by the interviewed clinicians (average scores were > 7). Drivers with the highest score were “effective on all AR symptoms” (average score of the whole sample: 9.1 ± 1.1) and “few/no side effects” (9.1 ± 1.0). On the other hand, cost-related aspects were associated with the lowest scores in all the specialty groups and with smaller percentage of physicians endorsing the positive response options (i.e. score > 9). For instance, only 36% of allergologists, 17% of ENTs, and 29% of GPs gave a positive answer to the item “refundable”.

### Patients’ sample characteristics

Clinical information of 2823 patients suffering from AR were collected; 909 patient records were retrieved by allergologists, 606 by ENTs, and 1308 by GPs (Table 2). Considering the whole sample, mean age of the majority of patients was < 44 (1902 patients, 67%) and 1414 patients (50.1%) were men. Analysis of patient clinical data confirmed that allergologists (327 patients, 36%) and ENTs (206 patients, 34%) visited more patients needing a new diagnosis than GPs (277 patients, 21%).

**Table 2:** Patients’ characteristics

Characteristic	Whole sample	Allergologists	ENTs	GPs	p-value
	N = 2823	N = 909	N = 606	N = 1308	
Age, years					*< 0.001*
18–24	630 (22.3%)	249 (27.3%)	148 (24.4%)	233 (17.8%)	
25–34	622 (22%)	219 (24%)	134 (22.1%)	269 (20.5%)	
35–44	650 (23%)	197 (21.6%)	139 (22.9%)	314 (24%)	
45–54	503 (17.8%)	149 (16.3%)	99 (16.3%)	255 (19.4%)	
> 55	412 (15%)	91 (10%)	85 (14%)	*236 (18%)*	
Disease duration, years	10 [5–19]	10 [4–15]	10 [5–20]*	10 [5–20]*	*< 0.001*
New diagnosis	810 (29%)	327 (36%)	206 (34%)	277 (21%)	*< 0.001*
AR causes					0.635
Graminaceous pollens	1450 (51.3%)	414 (45.5%)	314 (51.8%)	722 (55.1%)	
Tree pollens	873 (30.9%)	249 (27.3%)	165 (27.2%)	459 (35%)	
Grass pollens	722 (25.5%)	250 (27.5%)	168 (27.7%)	304 (23.2%)	
Dust/dust mites	1150 (40.7%)	352 (38.7%)	304 (50.1%)	494 (37.7%)	
Animal allergens	459 (16.2%)	130 (14.3%)	111 (18.3%)	218 (16.6%)	
Mould	262 (9.2%)	64 (7%)	69 (11.3%)	129 (9.8%)	
Cockroaches	14 (0.4%)	5 (0.5%)	2 (0.3%)	7 (0.5%)	
Other	5 (0.1%)	2 (0.2%)	0 (0%)	3 (0.2%)	
Concomitant asthma treatment	605 (89.8%)	238 (88.5%)	95 (88%)	272 (91.3%)	0.242

Patients’ data were retrospectively retrieved by the interviewed HCPs. Data are expressed as number of patients (%) ore median [25–75]. Chi squared test was used to investigate differences in the observed frequencies across specialty area. Kruskal–Wallis One Way Analysis of Variance on Ranks, All Pairwise Multiple Comparison Procedures (Dunn’s method): p < 0.05: * vs allergologists

Pollens were the more frequent cause of AR, followed by dust mites. More specifically, 1313 patients (46.5%) were allergic only to pollens, 439 (15.6%) only to dust, 222 (7.9%) to other causes, 849 (30.1%) to more than one cause. About a quarter of the cases (674 patients, 23.9%) suffered from concomitant asthma and the majority of these patients (605, 89.9%) took a specific drug for asthma treatment. Among asthmatic patients, 213 were only allergic to pollens, 117 only to dust, 36 only to other causes.

### AR clinical characteristics and disease severity according to HCPs

As shown in Table 3, symptoms reported by patients were similar across specialists. The most common symptoms involved upper respiratory tract: 2148 patients (76%) experienced congestion, 1931 (68.4%) sneezing, 1712 (60.6%) itchy nose, and 1677 (59.4%) runny nose. Ocular symptoms were likewise very common: itchy eyes affected 1085 patients (38.4%), red eyes 990 (35%), and watery eyes 983 (34.8%).

**Table 3:** Clinical characteristics of patients suffering from AR

Item	Whole sample	Allergologists	ENTs	GPs	p-value
	N = 2823	N = 909	N = 606	N = 1308	
AR symptoms					*< 0.001*
Congestion	2148 (76%)	686 (75.4%)	513 (84.6%)	949 (72.5%)	
Sneezing	1931 (68.4%)	669 (73.5%)	379 (62.5%)	883 (67.5%)	
Itchy nose	1712 (60.6%)	617 (67.8%)	305 (50.3%)	790 (60.3%)	
Runny nose	1677 (59.4%)	609 (66.9%)	369 (60.8%)	699 (53.4%)	
Itchy eyes	1085 (38.4%)	366 (40.2%)	131 (21.6%)	588 (44.9%)	
Red eyes	990 (35%)	303 (33.3%)	123 (20.2%)	*564 (43.1%)*	
Watery eyes	983 (34.8%)	307 (33.7%)	151 (24.9%)	*525 (40.1%)*	
Cough	651 (23%)	214 (23.5%)	104 (17.1%)	333 (25.4%)	
Itchy palate	461 (16.3%)	184 (20.2%)	100 (16.5%)	177 (13.5%)	
Difficult breathing	388 (13.7%)	127 (13.9%)	91 (15%)	170 (12.9%)	
Wheezing	369 (13%)	144 (15.8%)	35 (5.7%)	190 (14.5%)	
Sleep disorders/insomnia	193 (6.8%)	48 (5.2%)	46 (7.5%)	99 (7.5%)	
Irritability	143 (5%)	30 (3.3%)	22 (3.6%)	91 (6.9%)	
Chest tightness	117 (4.1%)	51 (5.6%)	*7 (1.1%)*	59 (4.5%)	
Fatigue	116 (4.1%)	32 (3.5%)	18 (2.9%)	66 (5%)	
Eczema	95 (3.3%)	29 (3.1%)	16 (2.6%)	50 (3.8%)	
AR severity					*< 0.001*
Moderate/severe	1906 (67.5%)	626 (68.8%)	456 (75.3%)	824 (62.3%)	
Mild	917 (32.4%)	283 (31.1%)	150 (24.7%)	*484 (37%)*	
Symptoms-related discomfort					*< 0.001*
Extremely bothersome (10–7)	1982 (70.2%)	604 (66.4%)	484 (79.9%)	894 (68.3)	
Moderately bothersome (6–5)	540 (19.1%)	188 (20.7%)	106 (17.5%)	246 (18.8%)	
Not bothersome (4–1)	301 (10.7%)	117 (12.9%)	16 (2.6%)	168 (12.8%)	
Discomfort scores according to severity					*< 0.001*
Moderate/severe	7.7 ± 1.3	7.7 ± 1.4	7.8 ± 1.2	7.7 ± 1.5	0.368
Mild	5.7 ± 1.9	5.4 ± 1.9	6.5 ± 1.3*#	5.6 ± 2.1	*< 0.001*

Patients’ clinical information was retrospectively retrieved by the interviewed HCPs. Data are expressed as mean ± SD or number of patients (%). Chi squared test was used to investigate differences in the observed frequencies across specialty area. One-way analysis of variance or two-way analysis of variance (factor A: specialty area, factor B: AR severity) followed by Tukey’s post hoc test. p < 0.001: * vs allergologists, # vs GPs

**Fig. 1:**
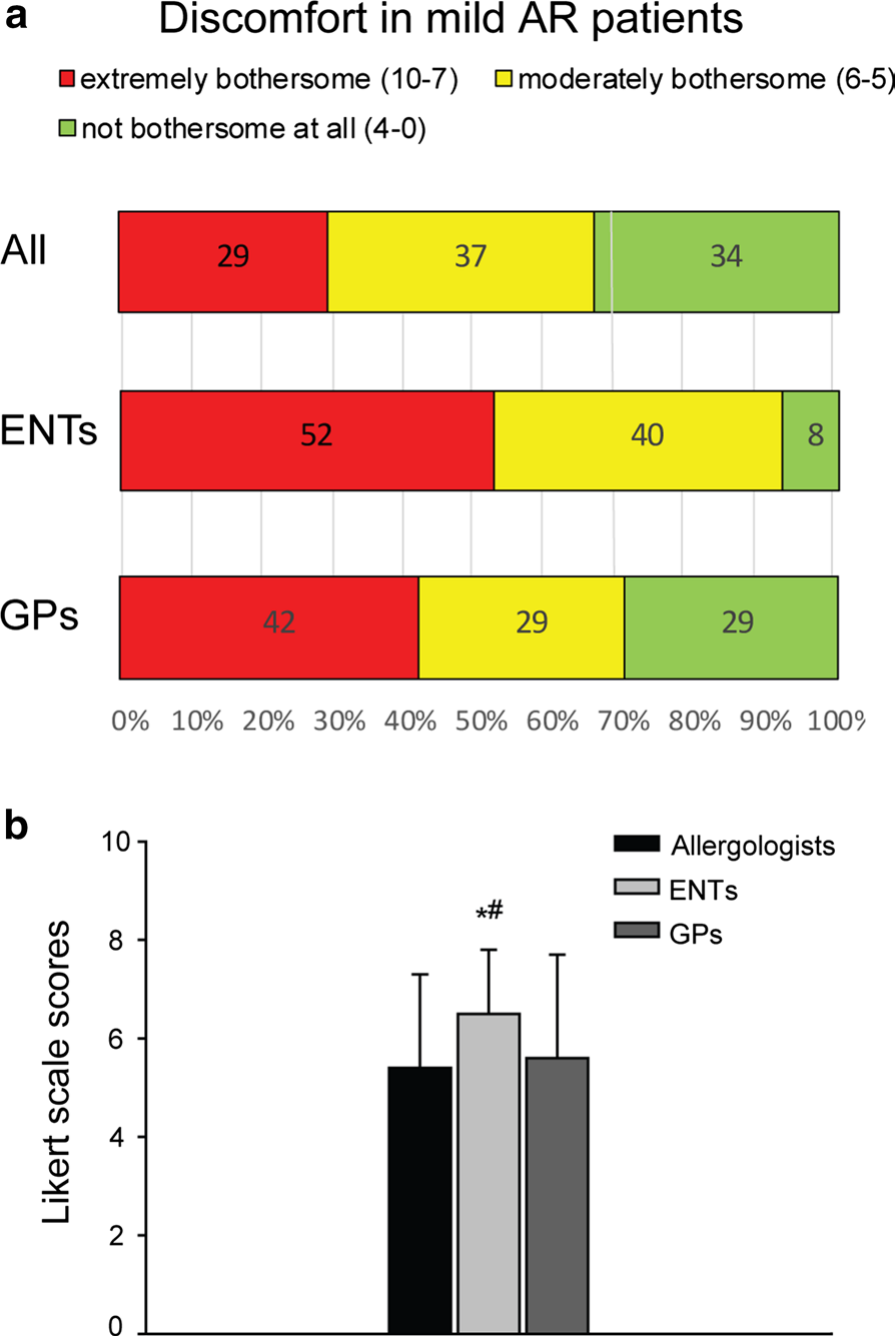
Symptoms-related discomfort experienced by patients suffering from mild AR. **a** Level of discomfort experienced by mild patients according to physicians. 10-point Likert scale: 1–4 = Not bothersome at all; 5–6 = moderately bothersome; 7–10 = Extremely bothersome. **b** Average scores of symptom discomfort. One way analysis of variance; all pairwise multiple comparison procedure (Tukey Test): p < 0.05: Asterisk vs allergologists, # vs GPs

From a physicians’ perspective, distribution of AR severity in the patients’ sample was: 1906 (67.5%) patients with moderate/severe AR and 917 (32.4%) patients with mild AR (Table 3). Concerning symptoms-related discomfort, physicians rated with high scores (> 7) the majority of their patients (1982 patients, 70.2%). Average scores of symptoms-related discomfort according to AR severity were 7.7 ± 1.3 for the moderate/severe group and 5.7 ± 1.9 for the mild group. Notably, about half of the patients assigned to the mild category were reported to suffer from extremely bothersome symptoms (Fig. 1a). ENTs rated these patients with higher scores relative to both allergologists and GPs (6.5 ± 1.3 vs 5.4 ± 1.9 and 5.6 ± 2.1, respectively, p < 0.001) (Fig. 1b).

Investigation of AR impact on patients’ professional life disclosed that about one-third of patients (1042 patients, 37.0%) reported reduced productivity due to AR (1338 patients with moderate/severe AR and 72 patients with mild, Additional file 1: Appendix S7, panel A). The majority of cases (703 patients, 67.7%) had a productivity impact score > 7. According to physicians, 551 patients (19.7%) complaints of work absenteeism due to AR, of whom 404 suffered from moderate/severe AR and 15 from mild AR (Additional file 1: Appendix S7, panel B).

### HCPs’ prescribing behaviour and AR therapy management

Table 4 reports the main prescription drivers based on patients’ characteristics. Overall, data were consistent with the previous analysis shown in Table 1. In fact, the item “effective on all AR symptoms” was the main prescription driver for the majority of patients (1533 patients on average, 54.3%), followed by “quick symptom relief” (1352 patients, 47.8%). On the other hand, “affordable price” and “refundable” were ranked low and were considered as relevant prescription drivers only for 13.4% and 8.7% of patients, respectively. Of note, “increased patient adherence” was the main prescription drivers for about 40% of patients visited by ENTs and GPs (40% and 35% of patients, respectively), while it was considered less significant by allergologists (28% of patients).

**Table 4:** AR therapeutic management by the interviewed physicians

Item	Whole sample	Allergologists	ENTs	GPs	p-value
	N = 2823	N = 909	N = 606	N = 1308	
Main prescription drivers					*< 0.001*
Effective on all AR symptoms	1533 (54.3%)	501 (55.1%)	335 (55.2%)	697 (53.2%)	
Quick symptom relief	1352 (47.8%)	429 (47.1%)	287 (47.3%)	636 (48.6%)	
Increased patient adherence	959 (33.9%)	*255 (28%)*	242 (39.9%)	462 (35.3%)	
Sustained efficacy	921 (32.6%)	307 (33.7%)	189 (31.1%)	425 (32.4%)	
Few/no side effects	911 (32.2%)	303 (33.3%)	*164 (27%)*	444 (33.9%)	
Effective with few drugs	849 (30%)	290 (31.9%)	198 (32.6%)	*361 (27.5%)*	
Easy to take	848 (30%)	228 (25%)	192 (31.6%)	428 (32.7%)	
Supported by scientific literature	470 (16.6%)	216 (23.7%)	127 (20.9%)	127 (9.7%)	
Affordable price	379 (13.4%)	137 (15%)	70 (11.5%)	172 (13.1%)	
Refundable	246 (8.7%)	60 (6.6%)	14 (2.3%)	*172 (13.1%)*	
Follow-up timing					< 0.001
< 12 months	86 (3%)	36 (4%)	24 (4%)	26 (2%)	
Every 12 months	1166 (41%)	427 (47%)	242 (40%)	497 (38%)	
Every 6 months	722 (26%)	272 (30%)	188 (31%)	262 (20%)	
< 6 months	865 (31%)	172 (19%)	157 (26%)	*536 (41%)*	
Treatment regimen					*< 0.001*
Monotherapy	1170 (41.4%)	303 (33.3%)	246 (40.5%)	*621 (47.4%)*	
Polytherapy	1653 (59.6%)	*606 (66.7%)*	360 (59.4%)	687 (52.5%)	

Data are expressed as number of patients (%). Chi squared test was used to investigate differences in the observed frequencies across specialty area

Polytherapy was the most common treatment strategy adopted by the interviewed physicians (1653 patients, 59.6%), while monotherapy was used in 41.4% of cases (1170 patients) (Table 4). Allergologists more often recommended polytherapy (606 patients, 66.7%, p < 0.001), while GPs adopted a monotherapy-based therapeutic approach for about half of their cases (621 patients, 47.4%, p < 0.001).

**Fig. 2:**
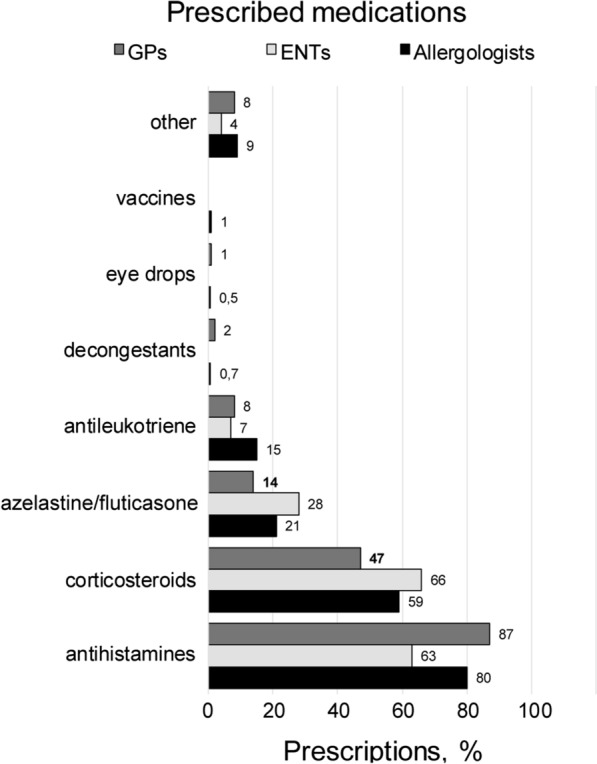
Distribution of drug prescriptions across specialty area. Data are expressed as % of prescriptions. Chi squared test was used to investigate differences in the observed frequencies across specialty area

With regard to prescribed medications, the most recommended classes of drugs were antihistamines and intranasal corticosteroids (2246 and 1549 prescriptions, respectively) followed by fixed-dose combination of intranasal azelastine/fluticasone (Aze/flu) (543 prescriptions) (Fig. 2). Compared to allergologists and ENTs, GPs less often recommended corticosteroids and fixed-dose combination of Aze/flu (p < 0.001, Fig. 2).

**Fig. 3:**
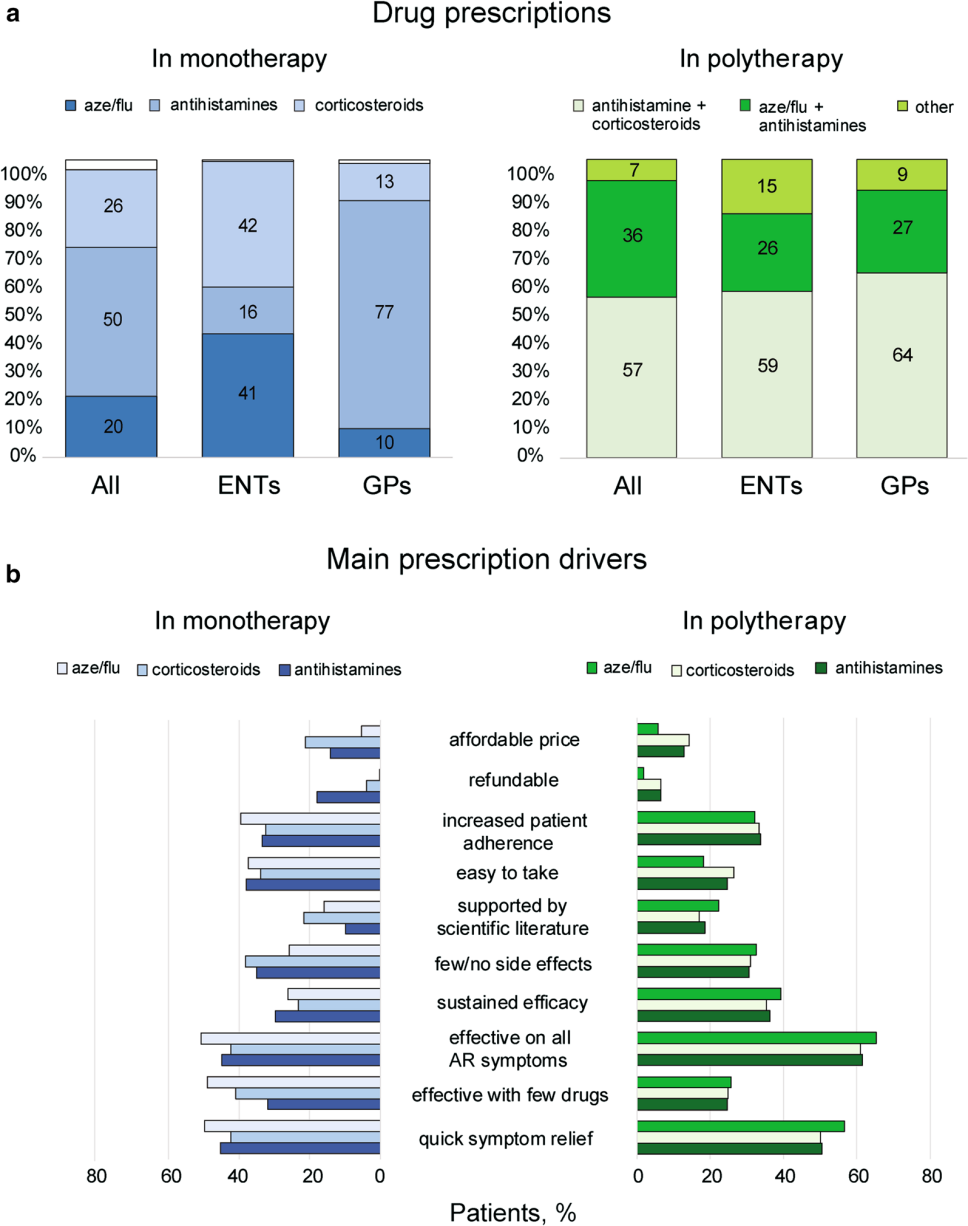
Monotherapy and polytherapy regimes: classes of drugs and main prescription drivers. **a** Use of the different classes of drugs within either monotherapy or polytherapy regimens. Monotherapy involves the use of a single drug, while polytherapy regimens are based on the use of different drugs. Data are expressed as % of patients. Chi squared test was used to investigate differences in the observed frequencies across specialty area. **b** Main prescription drivers in monotherapy and polytherapy regimes. Data are expressed as % of patients. Chi squared test was used to investigate differences in the frequencies across the different drugs. *Aze/flu* fixed-dose combination azelastine/fluticasone

Figure 3a shows the use of the different classes of drugs in either monotherapy or polytherapy regimens. Drugs preferentially used in monotherapy varied significantly across clinicians. Antihistamines were the most recommended medications by allergologists and GPs (50% of patients and 77% of patients, respectively), whereas ENTs more often prescribed corticosteroids (42%) and fixed-dose combination of Aze/flu (41%). Concerning polytherapy, loose combinations of antihistamines and intranasal corticosteroids were the most prescribed drugs (57%, 59%, and 64% of patients by allergologists, ENTs, and GPs, respectively). Aze/flu was largely used in monotherapy by ENTs (41%), while allergologists and GPs preferentially prescribed this drug in combination with antihistamines by (36% and 27%, respectively). Figure 3b displays the main prescription drivers adopted by HCPs in monotherapy and polytherapy regimes considering the most prescribed drugs, i.e. antihistamines, corticosteroids, and Aze/Flu.

In a further analysis focused on AR therapy management based on patients’ severity, treatment regimen and main prescription drivers were independently investigated for mild and moderate/severe patients (Additional file 1: Appendix S8). All the interviewed clinicians adopted different therapeutic approaches for mild and moderate/severe AR.

### HCPs’ opinions about patient adherence to treatment

**Fig. 4:**
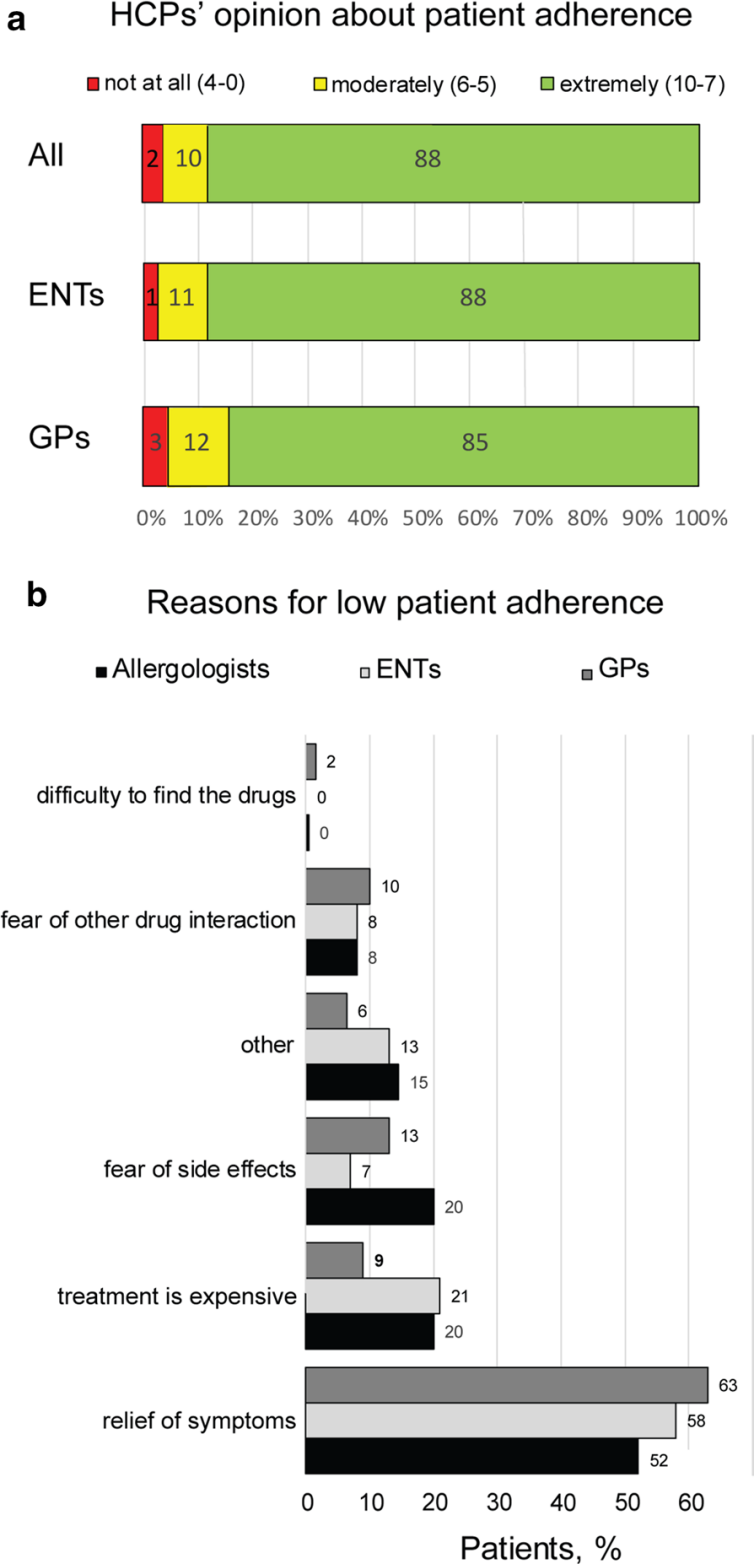
Physicians’ opinions about adherence to treatment of patients suffering from AR. **a** Diagram shows patients’ compliance according to the interviewed physicians. A 10-point Likert scale was used: 1 = Not at all; 10 = Extremely adherent. **b** Reasons for low adherence from clinicians’ perspective. Data are expressed as % of patients. Chi squared test was used to investigate differences in the observed frequencies across specialty area

Physicians believe that the majority of the patients (88% of patients with score > 7) has good adherence to treatment, even in the cases of severe AR (Fig. 4a). In HCPs’ opinion the main reasons for low patient compliance were “relief from the symptoms” and “treatment cost” (Fig. 4b).

## Discussion

The present survey investigated the current clinical practice scenario of AR management in Italy. In addition to provide an extensive description of Italian HCPs’ prescribing behaviour, this study discloses clinicians’ perspective about patients’ symptom discomfort and adherence.

AR is characterized by substantial medical and social burden with high use of healthcare resources worldwide [5, 6, 11, 34]. In addition, this disorder is associated with absenteeism from work, reduced productivity, and poor school performance [34, 35]. Recent studies indicate not only a global increase in the AR prevalence [3, 6, 36], but also high rates of underdiagnosis [3] and inadequate treatment [22].

In our survey, allergologist emerged as the main reference specialist for the disease in Italy, followed by ENTs. GPs visited more cases suffering from mild AR compared to both allergologists and ENTs. Prescription attitude was similar between HCPs. Attributes related to medication efficacy, safety, and patient adherence were considered more relevant prescription drivers than ease of use and cost-related items.

Consistent with previous Italian studies [31, 32], the most prescribed drugs were antihistamines and intranasal corticosteroids. A novel data disclosed by our survey is that allergologists and ENTs recommended fixed-dose combination intranasal Aze/flu to about 20% of the patients they visited. It is well-established that intranasal corticosteroids provide a more effective control of symptoms than antihistamines but their effect is relatively slow (hours) [18]. Fixed-dose combination of intranasal fluticasone propionate and azelastine hydrochloride was shown to be more efficacious than intranasal corticosteroid monotherapy [37–42] and it offers the additional benefit of faster relief of symptoms (minutes) [39, 40, 43]. This drug is also indicated when monotherapy with either intranasal antihistamines or corticosteroids do not adequately control the symptoms of AR [39, 41, 42, 44]. Of note, randomized clinical trials showed that fixed-dose formulation is more effective than loose combinations of corticosteroids and antihistamines in patients with moderate/severe seasonal AR [44]. The newest ARIA guidelines based on both Grading of Recommendations Assessment, Development and Evaluation (GRADE) and real-world evidence (RWE) confirm and emphasize efficacy of fixed-dose combination of intranasal Aze/flu for both nasal and ocular symptom relief and recommend the use of this drug as first line therapy for AR patients [45]. Our analysis showed that fixed-dose combination intranasal Aze/flu was used in both monotherapy and polytherapy regimens, with significant differences across clinicians. In fact, Aze/flu was preferentially used in monotherapy by ENTs, whereas it was more frequently recommended in polytherapy regimens by allergologists and GPs. This latter therapeutic strategy involved the simultaneous use of Aze/flu mainly together with antihistamines (ebastine, desloratine, bilastine). Assessing the risk of therapeutic duplication in patients suffering from AR is a crucial question that requires specific investigation.

A remarkable finding of the present survey is that AR severity is underestimated by physicians, irrespective of the specialty area in which they operate. In fact, about half of the patients assigned to the mild class of severity actually experienced particularly bothersome symptoms. This observation is consistent with data reported by the European survey carried out in Germany, France, Italy, Spain, and UK, in which clinicians not only underestimated the severity of disease but also misdiagnosed the nature and discomfort of symptoms [12]. As a correct classification of symptom frequency and severity is essential to select the best treatment option for each patient [13, 18, 46], an inaccurate patients’ allocation to severity categories can significantly affect AR therapeutic management. The results provided by our analysis of AR pharmacological management according to patients’ severity further supports this concept. Indeed, patients assigned to moderate/severe AR were preferentially recommended a polytherapy-based approach rather than a monotherapy regimen. Based on this, we can speculate that some of the patients improperly assigned to the mild category were undertreated. AR undertreatment and inadequate management have been extensively documented [20–22], suggesting that this disease is still trivialized in some cases [3, 22, 23].

With regard to HCPs’ opinions about patient adherence, our investigation disclosed that clinicians believe all the patients are compliant, even in the cases of severe AR. This perception does not reflect the real scenario of patients’ adherence in the AR settings. In fact, it is widely accepted that adherence in AR patients is very low [25, 26, 47, 48]. A recent study, in which compliance was assessed in a real-life setting using a mobile phone App, confirmed that about 70% of the recruited European AR patients are non-adherent to medications [26]. HCPs’ misperception of patient adherence in our sample could be partly due to low frequency of follow-up visits (once a year) and to a poor patients-clinicians communication [12, 49].

According to the interviewed physicians, the main cause of low compliance was relief of AR symptoms, followed by cost-related issues. Lack of efficacy, adverse effects, treatment duration, and costs are generally associated with lower compliance [50]. Patient satisfaction with treatment likewise appears to be a relevant factor in determining compliance, even if its contribution still needs to be elucidated. In fact, many researchers reported that dissatisfaction with treatment may cause non-adherence to therapy [51–53], whereas more recent studies revealed that patients discontinue their treatment when they felt better [47, 54]. In contrast to guidelines recommending the use of multiple drugs to achieve symptom control [45], recent data indicated that most patients experience poor symptom control with increasing medications [26, 55]. Hence, the use of single drug-based therapy could substantially ameliorate patient compliance. Finally, concerning drug cost, it is widely accepted that affordability of prescription medication has a role in therapy persistence [25]. Of interest, clinicians recruited in our survey did not consider cost issues as relevant prescription drivers.

## Conclusions

AR still represents a significant health problem because of the high burden of symptoms and the significant impact on patients’ QoL. The various available clinical guidelines state that accurate diagnosis, thorough patient evaluation, and adequate follow-up monitoring are a prerequisite to ensure optimal patient care.

The present research showed severity of AR symptoms is underestimated by Italian physicians, regardless of the specialty area in which they operate. This could lead to inadequate control of the disease. In addition, HCPs are not fully aware of the poor adherence to treatment.

These findings suggest that further efforts should be made to promote physicians’ adherence to clinical guidelines in order to improve AR management in Italy. Design of educational interventions for both GPs and specialists could improve characterization of the disease, help clinicians in the selection of the best treatment option, and promote a better patient-physician communication on the nature, severity, and impact of symptoms.

## Supplementary information

**Additional file 1.** Supplementary methods and results.

## Data Availability

The findings of the survey are available from Doxa Pharma S.r.l. However, these data were used under license and consequently they are not publicly available. Data are available from the authors upon reasonable request and with permission of Doxa Pharma S.r.l.

## Abbreviations

AR: Allergic rhinitis
ARIA: Allergic Rhinitis and its Impact on Asthma
Aze/flu: Azelastine/fluticasone
ENT: Ear, nose and throat
GPs: General practitioners
GRADE: Grading of Recommendations Assessment, Development and Evaluation
HCPs: Healthcare professionals
QoL: Quality of life
RWE: Real-world evidence
