# Supplementary material for: Physicians’ prescribing behaviour and clinical practice patterns for allergic rhinitis management in Italy
Source: Clin Mol Allergy. 2020 Nov 3;18:20. doi: 10.1186/s12948-020-00135-4 (PMC7640419; doi:10.1186/s12948-020-00135-4)
Supplement: Supplementary file 1 — Additional file 1. Supplementary methods and results. [file 12948_2020_135_MOESM1_ESM.docx]

Appendix

# Outline

[Outline 1](#_Toc23090482)

[Methods 2](#_Toc23090483)

[Appendix S1. Prescription drivers 2](#_Toc23090484)

[Appendix S2. Symptoms-related patient discomfort and AR severity classification 3](#_Toc23090485)

[Appendix S3. Therapy management 4](#_Toc23090486)

[Appendix S4. Prescription drivers based on patients’ characteristics 7](#_Toc23090487)

[Appendix S5. Patients’ adherence to treatment 8](#_Toc23090488)

[Appendix S6. AR impact on patients’ working life 9](#_Toc23090489)

[Results 10](#_Toc23090490)

[Appendix S7. AR impact on patients’ working life 10](#_Toc23090491)

[Appendix S8. AR therapy management based on patients’ severity 11](#_Toc23090492)

# Methods

## Appendix S1. Prescription drivers

S10. When you have to choose an allergic rhinitis treatment for your patients, how important are each of the aspects below?

Ratings occur along a 10-point Likert scale (1= Not important at all; 10=Extremely important).

1. That it works fast on the symptoms

2. That it has a price that is affordable for the patient

3. That it is has an easy form of administration

4. That it is effective with the smallest number of drugs

5. That it is supported by valid scientific literature

6. That it is effective on all allergic rhinitis symptoms

7. That it has long- lasting efficacy

8. That it doesn’t cause side effects

9. That it ensures better patient compliance

10. That it is refundable

## Appendix S2. Symptoms-related patient discomfort and AR severity classification

P6. How bothersome are this patient’s allergic symptoms?

Ratings occur along a 10-point Likert scale (1-4= Not bothersome at all; 5-6=moderately bothersome; 7-10=Extremely bothersome).

P7. What is the level of severity of this patient’s allergic rhinitis now?

1. Mild

2. Moderate/severe

3. Severe

## Appendix S3. Therapy management

P14. What treatment specific for ALLERGIC RHINITIS did you prescribe to this patient?

1. One drug only

2. Several drugs in combination, to be taken in the same period

3. Several drugs to be taken consecutively (i.e. one in a specific period and one in the following period)

4. Several drugs, some in cycles/in an ongoing manner and some as needed, in the same period

P15. Please indicate the drug/drugs for ALLERGIC RHINITIS you prescribed to this patient:

A-ANTIHISTAMINES

1. Aerinaze (desloradatina) – oral

2. Aerius (desloradatina) – oral

3. Allergodil (azelastina) – nasal spray

4. Allerwet (levocetiriziana) oral

5. Allespray (azelastina) – nasal spray

6. Ayrinal (bilastina) – oral

7. Azomyr (desloradatina) – oral

8. Cerchio (ceterizina) – oral

9. Ceteris (ceterizina) – oral

10. cetirizina – oral

11. Clarityn (loratadina) – oral

12. Clever (ebastina) – oral

13. Dasselta (desloradatina) – oral

14. desloratadina – oral

15. ebastina – oral

16. Efestad (desloradatina) – oral

17. Fexallegra (fexofenadina) – oral

18. Fixodin (fexofenadina) – oral

19. Formistin (ceterizina) – oral

20. Fristamin (loratadina) – oral

21. Kestine (ebastina) – oral

22. Levoreact (levocabastina)- nasal spray

23. Levostab (levocabastina)- nasal spray

24. levocetirizina

25. Mizollen (mizolastina) – oral

26. Mostaxyl (rupatadina) – oral

27. Neoclarityn (desloradatina) – oral

28. Netan (ebastina) – oral

29. Olisir (bilastina) – oral

30. Pafinur (rupatadina) – oral

31. Reactine (ceterizina+pseudoefedrina) – oral

32. Rinazina antiallergica (azelastina) – nasal spray

33. Robilas (bilastina) – oral

34. Rupafin (rupatadina) – oral

35. Stamidix (ceterizina) – oral

36. Suspiria (ceterizina) – oral

37. Telfast (fexofenadina) – oral

38. Tinset (oxatomide) - oral

39. Xyzal (levocetirizina) oral

40. Zirtec (ceterizina) – oral

41. Zolistam (mizolastina) – oral

B- CORTICOSTEROIDS

1. Aircort (budesonide) – nasal spray

2. Avamys (fluticasone) – nasal spray

3. Bentelan (betametasone) – nasal spray

4. Brusonex (mometasone) – nasal spray

5. Eltair (budesonide) – nasal spray

6. Flixonase (fluticasone) – nasal spray

7. Flixotide (fluticasone) – nasal spray

8. Fluspiral (fluticasone) – nasal spray

9. fluticasone – nasal spray

10. Nasofan (fluticasone) – nasal spray

11. Nasonex (mometasone) – nasal spray

12. Rinelon (mometasone) – nasal spray

13. Rinoclenil (beclomethasone dipropionato) – nasal spray

14. Zhekort (mometasone) – nasal spray

15. mometasone – nasal spray

C - CORTICOSTEROIDS+ANTIHISTAMINES

1. Dymista (azelastina cloridrato + fluticasone propionato) – nasal spray

D -DECONGESTANTS

1. Actidue (pseudoefedrina cloridrato) - oral

2. Actifed (pseudoefedrina cloridrato) – oral

3. Actigrip (pseudoefedrina cloridrato) – oral

4. Naristar (pseudoefedrina cloridrato) - oral

5. Narixan (pseudoefedrina cloridrato) - oral

6. Nurofen influenza e raffreddore (pseudoefedrina cloridrato) - oral

7. Vicks Flu-action (pseudoefedrina cloridrato) - oral

E - ANTILEUKOTRIENE

1. Airing (montelukast)- oral

2. Montegen (montelukast) – oral

3. Singulair (montelukast)- oral

F - OTHER DRUGS

1. Atem (ipratropio bromuro) – nasal spray

2. Atrovent (ipratropio bromuro) – nasal spray

3. Broncovaleas (salbutamolo solfato) – nasal spray

4. Kovinal (sodio nedocromile) – nasal spray

5. Lomudal (sodio cromoglicato) – nasal spray

6. Tilarin (sodio nedocromile) – nasal spray

7. Ventolin (salbutamolo) - nasal spray

P22. Every how often, more or less, do you see this patient for check-ups for allergic rhinitis?

1. Every _______ months

2. Once a year

3. Every _____ years

## Appendix S4. Prescription drivers based on patients’ characteristics

P17. What are the main reasons you prescribed this treatment, overall, to this patient? Please select the main 3 reasons

1. It works fast on the symptoms

2. It has a price that is affordable for the patient

3. It has an easy form of administration

4. It is effective with the smallest number of drugs

5. It is supported by valid scientific literature

6. It is effective on all allergic rhinitis symptoms

7. It gives long- lasting efficacy

8. It has few/no side effects

9. It ensures better patient compliance

10. It is refundable

## Appendix S5. Patients’ adherence to treatment

P19. In your opinion, how compliant will this patient be and follow the treatment for its entire duration?

Ratings occur along a 10-point Likert scale (1= Not at all; 10=Extremely).

P20. In your opinion, why might this patient not be completely compliant

1. Because the symptoms will be less bothersome and he will feel better

2. Because the treatment is expensive

3. Because it isn’t easy to find the drugs

4. Because he fears the possible side effects

5. Because he fears possible interaction with other drugs

6. Other reason

## Appendix S6. AR impact on patients’ working life

P8. Does this patient complain for productivity impact due to Allergic Rhinitis?

1. Yes

2. No

P9. And which level of productivity impact due to Allergic Rhinitis can we consider? Ratings occur along a 10-point Likert scale (1= No productivity impact at all; 10=Extreme productivity impact).

P10. Does this patient complain for absenteeism at work?

1. Yes

2. No

P11. How many days per year of absenteeism at work due to Allergic Rhinitis?

1. 1-5 days

2. 6-10 days

3. 11-15 days

4. >15 days

# Results

## Appendix S7. AR impact on patients’ working life

Panel A

|  |  | **Whole sample** |  | **Allergologists** | **ENTs** | **GPs** |  |
| --- | --- | --- | --- | --- | --- | --- | --- |
| **Item** | | **N=1038** |  | **N=364** | **N=230** | **N=471** | **p value** |
| **Reduced productivity** | |  |  |  |  |  | 0,346 |
|  | mild | 72 (6,9%) |  | 13 (4%) | 11 (5%) | 47 (10%) |  |
|  | moderate | 502 (48,4%) |  | 161 (48%) | 105 (46%) | 235 (50%) |  |
|  | severe | 736 (70,9%) |  | 275 (82%) | 145 (63%) | 315 (67%) |  |
| **Productivity impact** | |  |  |  |  |  | 0,869 |
|  | extreme productivity impact (10-7) | 703 (67,7%) |  | 228 (68%) | 158 (69%) | 315 (67%) |  |
|  | moderate productivity impact (6-5) | 256 (24,7%) |  | 84 (25%) | 59 (26%) | 113 (24%) |  |
|  | no productivity impact at all (4-0) | 77 (7,4%) |  | 23 (7%) | 11 (5%) | 42 (9%) |  |
| **Productivity impact scores** | | 7.0±1.9 |  | 7,0±1,7 | 7,0±1,5 | 6,6±1,7 | 0,256 |

Data are expressed as mean±SD or number of patients (%). Chi-squared test was used to investigate differences in the observed frequencies across specialty area. One-way Analysis of Variance was used to investigate differences between ratings across specialty area.

Panel B

|  |  | **Whole sample** |  | **Allergologists** | **ENTs** | **GPs** |  |
| --- | --- | --- | --- | --- | --- | --- | --- |
| **Item** | | **N=551** |  | **N=155** | **N=109** | **N=288** | **p value** |
| **Work absenteeism** | | |  |  |  |  | 0,407 |
|  | mild | 15 (2,7%) |  | 1 (1%) | 2 (2%) | 11 (4%) |  |
|  | moderate | 140 (25,4%) |  | 30 (20%) | 22 (21%) | 86 (30%) |  |
|  | severe | 264 (47,9%) |  | 83 (54%) | 37 (34%) | 143 (50%) |  |
| **Lost working days** | | |  |  |  |  | 0,332 |
|  | >15 d | 69 (12,6%) |  | 15 (10%) | 10 (10%) | 43 (15%) |  |
|  | 11-15 d | 78 (14,1%) |  | 15 (10%) | 10 (10%) | 51 (18%) |  |
|  | 6-10 d | 178 (32,3%) |  | 54 (35%) | 34 (32%) | 89 (31%) |  |
|  | 1-5 d | 225 (40,9%) |  | 69 (45%) | 52 (48%) | 103 (36%) |  |

Data are expressed as % of patients. Chi-squared test was used to investigate differences in the observed frequencies across specialty area.

## Appendix S8. AR therapy management based on patients’ severity

|  |  | **Allergologists** | | | **ENTs** | | | **GPs** | | |
| --- | --- | --- | --- | --- | --- | --- | --- | --- | --- | --- |
|  |  | **Mild** | **Moderate/severe** | **p value** | **Mild** | **Moderate/severe** | **p value** | **Mild** | **Moderate/severe** | **p value** |
|  |  | **N=283** | **N=626** |  | **N=150** | **N=456** |  | **N=484** | **N=824** |  |
| **Treatment regimen*** | |  |  | **p<0.001** |  |  | **p<0.001** |  |  | **p<0.001** |
|  | monotherapy | **184 (65%)** | **119 (19%)** |  | **85 (56,6%)** | **161 (35,3%)** |  | **357 (73,7%)** | **264 (32%)** |  |
|  | concomitant polytherapy | **74 (26,1%)** | **407 (65%)** |  | **45 (30%)** | **227 (49,7%)** |  | **103 (21,2%)** | **406 (49,2%)** |  |
|  | sequential polytherapy | 5 (1,7%) | 21 (3,3%) |  | 6 (4%) | 14 (3%) |  | **4 (0,8%)** | **25 (3%)** |  |
|  | polytherapy (continuous + as-needed) | **20 (7%)** | **79 (12,6%)** |  | 14 (9,3%) | 54 (11,8%) |  | **20 (4,1%)** | **129 (15,6%)** |  |
| **Main prescription driver** | |  |  |  |  |  |  |  |  |  |
|  | efficacy on rhinitis symptoms | **52 (18,3%)** | **188 (30%)** | **p<0.001** | 36 (24%) | 107 (23,4%) | **p<0.001** | 122 (25,2%) | 217 (26,3%) | **p=0,047** |
|  | quick symptom relief | 49 (17,3%) | 109 (17,4%) |  | **19 (12,6%)** | **85 (18,6%)** |  | 78 (16,1%) | 157 (19%) |  |
|  | sustained efficacy | **24 (8,4%)** | **78 (12,4%)** |  | **7 (4,6%)** | **57 (12,5%)** |  | **33 (6,8%)** | **79 (9,5%)** |  |
|  | few/no side effects | 28 (9,8%) | 41 (6,5%) |  | 11 (7,3%) | 24 (5,2%) |  | 38 (7,8%) | 50 (6%) |  |
|  | more efficacy with few drugs | **44 (15,5%)** | **64 (10,2%)** |  | 21 (14%) | 63 (13,8%) |  | 62 (12,8%) | 90 (10,9%) |  |
|  | increased adherence | 24 (8,4%) | 49 (7,8%) |  | **28 (18,6%)** | **48 (10,5%)** |  | 43 (8,8%) | 89 (10,8%) |  |
|  | easy to take | **25 (8,8%)** | **21 (3,3%)** |  | **21 (14%)** | **28 (6,1%)** |  | **53 (10,9%)** | **56 (6,7%)** |  |
|  | supported by scientific literature | 25 (8,8%) | 50 (7,9%) |  | **4 (2,6%)** | **33 (7,2%)** |  | 11 (2,2%) | 28 (3,3%) |  |
|  | affordable price | 3 (1%) | 18 (2,8%) |  | 2 (1,3%) | 9 (1,9%) |  | 19 (3,9%) | 26 (3,1%) |  |
|  | refundable | 9 (3,1%) | 8 (1,2%) |  | 1 (0,6%) | 2 (0,4%) |  | 25 (5,1%) | 32 (3,8%) |  |

*Monotherapy involves the use of a single drug, while polytherapy regimens are based on the use of different drugs. In particular, polytherapy includes the following regimens: concomitant polytherapy, in which different drugs are simultaneously used; sequential polytherapy, in which the use of a specific drug is sequential to the use of another drug; polytherapy, in which different drugs, of which some continuously and some as-needed, are used.

Data are expressed as % of patients. Chi-squared test was used to investigate differences in the observed frequencies between mild and moderate/severe classes.
